# Supplementary material for: Habit degradation strategies promote faster early reductions in unhealthy snacking habit strength in intensive longitudinal randomised controlled trial
Source: Commun Psychol. 2026 Mar 4;4:67. doi: 10.1038/s44271-026-00432-9 (PMC13076195; doi:10.1038/s44271-026-00432-9)
Supplement: Supplementary file 2 — Supplementary Information [file 44271_2026_432_MOESM2_ESM.pdf]

Supplementary information for "Habit degradation strategies promote faster early reductions in unhealthy snacking habit strength in intensive longitudinal randomised controlled trial"

Authors: Robert Edgren[1], PhD, Dario Baretta[1], PhD, & Jennifer Inauen[1], PhD

[1]Department of Health Psychology and Behavioral Medicine, University of Bern, Bern, Switzerland

Author note

Robert Edgren: robert.edgren@unibe.ch; https://orcid.org/0000-0003-1901-5571

Dario Baretta: https://orcid.org/0000-0002-2866-3290

Jennifer Inauen: https://orcid.org/0000-0002-7884-3222

Table of Contents

1. Supplementary methods 4
1.1 Sample size justification 4
1.2 Reward message pilot study 5
1.2.1 Methods 5
1.2.2 Results 7
1.3 Procedure 8
1.3.1 Randomisation 8
1.3.2 Reimbursement 9
1.3.3 Fixed interval-contingent data collection 9
1.4 Experimental manipulation 9
1.4.1 Experimental group specific instructions 9
1.4.2 App features 18
1.5 Methods for intervention fidelity and manipulation check 19
1.6 Data analysis 20
1.6.1 Event contingent data processing 20
1.6.2 Main analysis assumption testing 20
1.7 Statistical software 21
1.8 Deviation from protocol 21
2. Supplementary notes 22
2.1 Supplementary notes 1: Participant retention and habit strength observations 22
2.2 Supplementary notes 2: Within-person habit degradation trajectories and outcomes extracted 24
2.3 Supplementary notes 3: Main analysis 27
2.4 Supplementary notes 4: Intervention fidelity and manipulation check 28
2.5 Supplementary notes 5: Sensitivity analyses 29
Supplementary references 37

List of supplementary tables

Table S1 | Intrinsic Motivation Inventory items..6

Table S2 | Example reward messages...19

Table S3 | Descriptive statistics of observed habit strength and indicators of habit degradation by allocated intervention group..26

Table S4 | Pairwise comparisons from logistic regression results.........................27

Table S5 | Descriptive statistics of variables used in sensitivity analyses.............30

Table S6 | Estimated marginal means and test statistics of sensitivity analyses with magnitude of change as outcome (H1.1-.3)..31

Table S7 | Estimated marginal means and test statistics of sensitivity analyses with likelihood of reaching asymptote as outcome (H1.4-.6)..35

Table S8 | Estimated marginal means and test statistics of sensitivity analyses with rate of change as outcome (H2.1-.3)...36

List of supplementary figures

Fig. S1 | Screenshots from smartphone app..18

Fig. S2 | Participant flow chart..22

Fig. S3 | Proportion of missing daily habit strength observations..........................23

Fig. S4 | GAMs and asymptotic models that did not meet set criteria..................25

1. Supplementary methods

1.1 Sample size justification

A total of 307 participants were aimed to be recruited, with 43 participants per group for the six intervention groups and 49 participants for the control group. The rationale for the sample size was based on an ANCOVA power analysis, which aimed to detect a main effect for intervention group (three strategies) and reward in predicting average habit strength at the study's conclusion. The smallest effect size of interest was defined as a 0.5 difference in habit strength (on an SRBAI scale ranging from 0-4) between groups. Specifically, an a priori ANCOVA (3x2 factorial design) was conducted with a Bonferroni-corrected alpha value of 0.05/6 and a beta value of 0.8. Based on data from our previous study[1], the standard deviation was presumed to be 1, and the R-squared value of initial habit strength (as a covariate) was presumed to be 0.12. Consequently, the smallest effect size of interest was considered small-to-medium (Cohen's *d* = 0.50). Power analyses were conducted using exact estimation with the R package Superpower[2]. This analysis concluded that 180 participants were required (30 participants per group across six groups). It was further verified that for comparisons involving the control group with all experimental groups combined, the control group required an additional four participants (*N*=34) to achieve 80% power to detect a group difference of 0.5 in habit strength (with an alpha of 0.05, standard deviation of 1, and R-squared of 0.12). Therefore, the total required sample size was 214 participants. Using an expected retention rate of 70% and rounding upwards to ensure equally sized groups, a total sample size of 307 was required for recruitment. For hypotheses concerning the rate of change, the previously described power analysis was considered sufficient to detect small-to-medium effect sizes (Cohen's *d* = 0.50). The estimated retention rate was based on a previous study[1], where the retention rate was 60%; however, planned procedural changes, such as shorter questionnaires and providing compliance information during study participation, aimed to improve this retention rate by 10%. Substantial interindividual differences in habit strength trajectories were expected based on our previous

study[1]. Due to these interindividual differences, power analysis was not conducted for analyses based on asymptotic modelling. Specifically, it was not expected that decreasing habit strength trajectories would be adequately described by an asymptotic trend across the entire sample, which made reliable power analysis difficult for such cases.

1.2 Reward message pilot study

1.2.1 Methods

An initial list of 107 reward messages were created by the research team. The reward messages were created such that they contained, to varying degrees, congratulating text on accomplishment, and text intending to address the participant's competence and autonomy. For generating these reward messages, ChatGPT[3] (version GPT-3.5) was used.

Subsequently, a cross-sectional pilot study was conducted to get initial feedback on how these reward messages are perceived. For evaluating perceived reward, each message was evaluated with adapted items from selected subscales of the Intrinsic Motivation Inventory (IMI)[4,5]. See Table S1. for items used. 3 items were selected for each of the Interest/Enjoyment, Perceived competence, and Perceived choice subscales. These three subscales were of primary interest, as they correspond to liking, competence and autonomy. Of note, 1 item from the Perceived Choice subscale was a novel addition: "Reading this message, I feel like I am obliged to change my snacking behaviour" (reverse scored). Additionally, 2 items from the Value/Usefulness subscale (as perceived value could relate to any psychological need). Also, 1 item not related to the IMI was included, namely "This message feels rewarding to me". All items were assessed on a 5-point Likert scale (scored 1-5). Higher scores indicated stronger agreement with the statements, indicating higher perceived reward (except for the previously noted perceived choice item that was reversed scored). As such, in total 12 items were used to evaluate each reward message. Lastly, participants could give open feedback for each reward message.

Table S1 | Intrinsic Motivation Inventory items used in reward message pilot study.

Subscale	Item wording
Interest/Enjoyment	This message was quite enjoyable.
	This message made me feel good.
	I found this message interesting.
Perceived competence	Reading this message, I feel a little more competent in changing my unhealthy snacking.
	Reading this message, I think I am pretty good at changing my snacking behaviour.
	Reading this message, I feel more satisfied with my performance at changing my snacking behaviour.
Perceived Choice	Reading this message, I feel reassured that changing my snacking behaviour is my own choice.
	Reading this message, I feel more strongly that I want to change my snacking behaviour.
	Reading this message, I feel like I am obliged to change my snacking behaviour.
Value/Usefulness	This message has some value to me.
	I would find it useful to receive this message again in the future.

Note. Item wording is translated from German

Pilot study participants were instructed to imagine themselves taking part in a study where they intend to reduce unhealthy snacking, and that they receive the positive feedback messages in response to successfully avoiding eating unhealthy snacks. Each participant evaluated 23 reward messages. Respondents could enter a raffle to win 1 of 3 supermarket gift vouchers worth 50 CHF for taking part in the study. Data collection took place from January 6th to February 12th, 2024.

Reward messages with the highest mean score (>=3.5) across all items were qualitatively evaluated to identify commonalities and differences among these messages. Similarly, messages with low overall mean scores (<3.0) were evaluated to identify commonalities of content that was not appreciated. Additionally, the open responses participants provided were inspected to identify content that was appreciated or that needed revision. The mean score of reward messages for subscales Interest/Enjoyment, Perceived Choice and Perceived Competence were inspected to see how the messages address these different facets of reward.

Subsequently, a new set of 85 (1 unique message per study day from day 7 to 91) reward messages were generated. Here, initial reward messages that had high overall means were used as a reference to generate novel messages with similar content and structure. To ensure variability in the exact wording of reward messages ChatGPT was used to generate novel messages.

1.2.2 Results

In total, 272 participants fulfilled inclusion criteria and provided informed consent. Out of these 68 participants completed the entire survey (i.e. assessed 23 reward messages with 12 items each). An additional 83 participants provided incomplete responses to the survey (i.e. assessing at least some reward messages). Effectively, each reward message was evaluated with 146 to 336 responses (where 1 message was evaluated with a maximum of 12 items by 1 participant). The mean age of the sample that completed the survey (*N* = 68) was 42.9 years (*SD* = 15.4), and 55 participants (80%) identified with female gender, and 10 with male gender.

1.2.2.1 Quantitative findings. In total, 29 reward messages had a mean score of 3.5 or higher across all items (i.e. 0.5 points above scale midpoint). These messages tended to be 1 or 2 sentences in length, often containing over 7 words. These included messages that provided specific feedback to the performed behaviour, praising the participant for their achievement (e.g. "you have proven your assertiveness once again"), acknowledgment of progress made and milestones reached (e.g. "your progress over the last few weeks is really impressive" & "Day 10!"), and emphasizing perseverance (e.g. "You have clearly demonstrated your impressive resilience today!") and self-determination (e.g. "You are incredibly determined to keep your resolution"). Also, these messages encouraged maintaining progress (e.g. "keep it up!"). Interestingly, messages evaluated as more rewarding did not include emojis. 11 reward messages were scored below the scale midpoint (score below 3). These included short messages (1-2 words), and messages that did not explicitly address the performed behavior (e.g. "Simply fantastic!"). It seemed that messages that didn't empower individuals or highlight their agency

in decision-making tended to be perceived as less rewarding. All items were scored similarly across each IMI subscales interest/enjoyment, perceived competence, and perceived choice, and these subscale scores corresponded closely to the overall mean score.

1.2.2.2 Qualitative findings. Participants provided in total 313 open comments in reference to 101 messages. Among these, each reward message received 1-9 open comments. Suggestions for improvement include adding statements such as "well done", avoiding long and complicated sentences and usage of technical language, and encouragement to reflect on the reasons for behavioural change.

1.3 Procedure

1.3.1 Randomisation

The study app, "Habirupt" was developed by the research team in collaboration with the University of Bern Technology Platform for Research using the in-house "self-help" platform which supports app development. Participants were randomly allocated to one of 7 study groups using a restricted randomisation approach to ensure balanced group sizes. The allocation sequence was generated and implemented automatically within the self-help platform using a computerized random number generator. In each allocation cycle, all 7 groups were initially available. The first participant in a cycle was assigned to a randomly chosen group, the second participant randomly assigned to one of the remaining 6 groups, and so forth until all 7 groups had received one participant. The process then repeated for subsequent cycles, ensuring that the distribution of participants across groups remained as even as possible throughout the study. Note that the group counter was initially set to six for all intervention groups and zero for the control group, to ensure that the control group would have 6 participants more than the intervention groups, as per the planned sample size. Consequently, the first 6 participants were assigned to control, after which allocation took place per the above restricted randomisation approach described above with a 1:1 ratio.

1.3.2 Reimbursement

Participants were informed they would receive CHF 120 for full study participation, with a minimum requirement of completing 4/7 end-of-day questionnaires each week. For incomplete participation, participants received CHF 9 per week when they had completed at least four end-of-day questionnaires.

1.3.3 Fixed interval-contingent data collection

The end-of-day questionnaires were estimated to take 2-3 minutes to complete. The end-of-day questionnaire was available in the app from 19:00 PM until 10:00 AM the following morning. The end-of-day questionnaire for Days 1-7 consisted of 15-16 multiple-choice items and two open-ended items. For Days 8-91, the end-of-day questionnaire comprised 22-23 multiple-choice items and one open-ended item.

1.4 Experimental manipulation

1.4.1 Experimental group specific instructions

Instructions during cue identification phase (all participants; days 1-6)
Sometimes we eat unhealthy snacks in response to signs we experience in our daily lives. We would now like to ask you to think about the personal triggers that you experience in your daily life at home and which cause you to consume unhealthy snacks. To do this, please pay attention to your unhealthy snacking behaviour when you are at home during the first 7 days of the study.<br>Both today and in the coming days, we will ask you in the evening what triggers you experienced at home on each day.<br><br>By **triggers**, we mean things that cause you to consume unhealthy snacks due to a habitual behaviour. For example, seeing the TV or entering the kitchen. There are many other possible triggers.<br><br>By **habitual behaviour** we mean a behaviour that has become automated due to constant repetition when a trigger occurs. Triggers can be objects, people, routines or times, for example. A strong habitual behaviour is then performed automatically when the trigger occurs. In order to break a habitual behaviour, it is therefore important to first find the trigger.<br><br>**Here are a few examples:**<br>- Anna eats a pastry during her afternoon coffee break. Personal trigger: Afternoon coffee break (routine).<br>- Anna sees the cookie tin in her kitchen cupboard and grabs a cookie straight away. Personal trigger: cookie tin (object).<br>- At 4 p.m. Anna sits down and eats a cookie. Personal trigger: 4 p.m. (time).

At the end of the first week, you will be instructed to choose a specific trigger that you frequently encounter in your everyday life at home. The goal will be to stop your unhealthy snacking behaviour for this cue for the duration of the study. This is the habit you will try to change.

Here are some more examples of triggers that participants have already mentioned:
- Seeing snacks in the kitchen
- Opening the fridge
- Being home alone
- being at home with someone who is eating snacks
- hunger; thirst
- stress
- Craving for something sweet
- Coffee break in the afternoon
- after lunch

***
Now we would like to ask you what triggers you experience in your everyday life at home that tempt you to consume unhealthy snacks. Writing down the triggers for your unhealthy snack consumption will help you to understand your habitual snacking behaviour.

Please write down the triggers you have experienced at home today.
Example: "Entering the kitchen, sitting on the sofa, ..."

[Open response]

Instructions for cue selection (all participants; day 7)

Welcome to the second part of today's survey!

Now it's time to choose a snacking habit that you would like to change during the study. Before doing so, we would like to explain to you once again what a habit is and how you can change habitual snacking behaviour. This understanding is important for participation in the study.

As a reminder:
By habitual behaviour, we mean behaviour that has become automatic due to constant repetition when a cue occurs. Cues can be objects, people, routines or times, for example. A strong habitual behaviour is performed automatically when the cue occurs. In order to break a habitual behaviour, it is therefore important to first identify the cue.

Here are a few examples:

Anna eats a pastry during her afternoon coffee break.
Personal cue: Afternoon coffee break (routine)

Anna sees the biscuit tin in her kitchen cupboard and grabs a biscuit straight away.
Personal cue: biscuit tin (object)

At 10 a.m. Anna sits down and eats a biscuit.
Personal cue: 10 a.m. (time)

When Anna’s friend Josephine arrives, they eat chocolate together.
Personal cue: friend (person)

Now it's your turn to define the cue for your snacking habit that you want to change. Remember, you should focus on this cue of your snacking habit during the 12-week study period.<br><br>Below we have compiled the cues that you have observed and reported in your diary over the past week.<br><br>You can now choose a cue that best fits the following criteria.<br><br>You are also welcome to choose a cue that is not listed here if something comes to mind that fits even better.<br><br>This will be the snacking habit that you try to change during the study.<br><br>**The cues you observed last week:**<br>{piped text of week 1 observed cues}<br><br>**Choose a cue...**<br>...that you encounter at home<br>...that you experience about once a day<br>...that usually tempts you to eat unhealthy snacks<br><br>**I choose the following cue:**<br>[open response]

**Control** group instructions (day 7)
**You were randomly selected to take part in the control condition of the study.**<br><br>From a research perspective, this is a very important role. Only with a control group will we be able to determine whether the investigated strategies are indeed effective to disrupt a habit.<br><br>**We therefore ask you to answer the evening questionnaires over the next 12 weeks and not actively try to change your unhealthy snacking habit.**<br><br>At the end of the study, you will be given access to all the materials that can help you to break your snacking habits in the future if you wish.<br><br>If you have any questions or comments, please feel free to contact us at habirupt.psy@unibe.ch.

Instructions for implementation intention formulation (**all intervention** groups; day 7)
Now it's time to decide how you're going to prevent your unhealthy snack consumption when your "{cue}" cue occurs.<br><br>**The key to changing a habit lies in the following steps:**<br><br>- First, you need to identify the cue that is causing the habitual behaviour. You have just completed this step!<br>- Second, you need to create a plan for how to overcome your habitual behaviour.

- Third, you need to define the preparatory measures you need to implement the plan.
- And fourth, you should consistently implement the plan when you encounter the cue.

In the following, we will support you step by step in changing your habits.

Instructions for implementation intention formulation (**Substitution** group; day 7)

Now we come to the second step for habit change: you create a plan for how to overcome your habitual behaviour.

Habit research suggests that replacing an old habitual behaviour with a new behaviour can help to successfully break the old habit.

Example: Anna wants to change her habit of eating a pastry with her coffee in the afternoon. She therefore plans to eat a fruit instead of a pastry (substitute behaviour).

Now it is up to you to decide which alternative you want to use to replace the unhealthy snack when your cue "{cue}" occurs. There are two different options:

[1] replace the unhealthy snack with a healthy one (e.g. fruit or nuts)
[2] Replace the unhealthy snack with something completely different (e.g. go for a walk)

***
If [1] was selected:

For your cue "{cue}", you have chosen to consume something else in place of the unhealthy snack.

Examples:
- eat fruit or nuts instead of an unhealthy snack
- drink unsweetened coffee, tea or water instead of an unhealthy snack

Now we would like you to formulate an if-then plan.

Scientific studies show that creating a precise if-then plan helps you to implement it in the future.

Importantly, an if-then plan should have the following format:

If I [insert your cue], **then I will** [insert your healthy snack].

Example: "If I drink a coffee in the afternoon, then I will eat an apple."

In this example, "drink coffee in the afternoon" is the cue and "eat an apple" is the replacement action.

***
If [2] was selected:

For your cue "{cue}", you have chosen to replace the unhealthy snack with something completely different.

Examples:
- go for a walk

- Doing sport
- reading
- brush your teeth

Now we would like you to formulate an if-then plan.

Scientific studies show that creating a precise if-then plan helps you to implement it in the future.

Importantly, an if-then plan should have the following format:

If I [insert your cue], **then I will** [insert your alternative activity].

Example: "If I drink a coffee in the afternoon, then I will read a book."

In this example, "drink coffee in the afternoon" is the cue and "read a book" is the alternative activity.

***
[1]&[2]:

Please now write down your if-then plan exactly according to this scheme.

Please note that you will keep to this plan for the next 12 weeks and cannot change it.
[open response]
***

If [1] was selected:

Now we come to the preparatory step for habit change:

Please think about the preparatory measures needed to implement your plan.

This could be, for example:
- buy the selected substitute product
- keep the replacement product in the right place (e.g. next to the sofa or in the kitchen)
- set a notification / reminder on your mobile device
- put a handwritten note where you can see it
- discuss your plans with people close to you
***

If [2] was selected:

Now we come to the preparatory step for habit change:

Please think about the preparatory measures needed to implement your plan.

This could be, for example
- setting a notification / reminder on your mobile device
- Put a handwritten note where you can see it
- Discuss your plans with people close to you
***

[1]&[2]:

What do you need to do to realise your plan? [open response]

Instructions for implementation intention formulation (**inhibition** group; day 7)
**Now we come to the second step for habit change: you create a plan for how** to overcome your habitual behaviour**.**<br><br>Habit research suggests that it can be helpful to inhibit the habitual behaviour when the cue occurs.<br><br>**Example:**<br>Anna wants to change her habit of eating a pastry with her coffee in the afternoon. She could plan to avoid eating the pastry by thinking about her goal of not eating pastries.<br><br>Now it's up to you to decide how you want to inhibit your unhealthy snack consumption when your cue "{cue}" occurs. There are several ways to do this:<br><br>[1] think of something motivating (e.g. your goals)<br>[2] think of the limits you set for yourself (e.g. that you do not eat any chocolate during the week)<br>[3] Redirect your attention (e.g. think about something else)<br><br>***<br>If [1] was selected:<br><br>**Think about how you want to motivate yourself.**<br><br>**Examples:**<br>- Think about your goal of not snacking<br>- telling yourself that you can resist the craving for snacks by simply waiting<br>- telling yourself that snacking will not improve the situation<br><br>**Now we would like you to formulate an if-then plan.**<br><br>Scientific studies show that creating a precise if-then plan helps you to implement it in the future.<br><br>**Importantly, an if-then plan should have the following format:**<br><br>**If I** [insert your cue], **then I will** [insert your motivating thought].<br><br>**Example:** "If I'm in the kitchen at home, then I will tell myself that I can resist the craving for snacks by simply waiting."<br><br>In this example, " in the kitchen at home" is the cue and "then I will tell myself that I can resist the craving for snacks by simply waiting" is the motivating thought.<br><br>***<br>If [2] was chosen:<br><br>**Think about how you want to set limits for yourself.**<br><br>**Examples**:<br>- not allowing yourself to look in the fridge<br>- only eat snacks on special occasions (e.g. on holidays)<br>- only eat snacks on certain days

Now we would like you to formulate an if-then plan.

Scientific studies show that creating a precise if-then plan helps you to implement it in the future.

Importantly, an if-then plan should have the following format:

If I [insert your cue], **then I will** [insert your limit].

Example: "If I'm in the kitchen late in the evening, I won't allow myself to open the fridge."

In this example, " in the kitchen late in the evening" is the cue and "then I will not allow myself to open the fridge" is the limit.

***
If [3] was chosen:

Think about how you can focus your attention elsewhere when your cue "{cue}" occurs.

Example:
- pause for a moment and focus on my breathing
- think about what was the funniest thing that happened today
- count backwards from 20

Now we would like you to formulate an if-then plan.

Scientific studies show that creating a precise if-then plan helps you to implement it in the future.

Importantly, an if-then plan should have the following format:

If I [insert your cue], **then I will** [insert your inhibiting action].

Example: "When I'm in the kitchen late in the evening, I will stop and focus on my breathing."

In this example, " in the kitchen late in the evening" is the cue and "then I will stop and focus on my breathing" is the inhibitory action.

***
[1]&[2]&[3]:

Please now write down your if-then plan exactly according to this scheme.

Please note that you can keep to this plan for the next 12 weeks and cannot change it.
[open response]

***
[1]&[2]&[3]:

Now we come to the third step for habit change:

Please think about the preparatory measures needed to implement your plan.

This could be, for example

- setting a notification / reminder on your mobile device
- Put a handwritten note where you can see it
- Discuss your plans with people close to you

What do you need to do to realise your plan? [open response]

Instructions for implementation intention formulation (**Reduced accessibility** group: day 7)

Now we come to the second step for habit change: you create a plan for how to overcome your habitual behaviour**.**

Habit research suggests that limiting the availability of a behaviour can help to break the habit. So, making sure to not have snacks available at home.

Example:
Anna wants to change her habit of eating a pastry with her coffee in the afternoon. She could plan to give away her existing pastries when she gets home tonight, and therefore will no longer have pastry at home.

Now we would like you to formulate an if-then plan to no longer have snacks available at home.

Scientific studies show that creating a precise if-then plan helps you to implement it in the future.

Importantly, an if-then plan should have the following format:

If I [insert when you will limit the availability of snacks], **then** [insert how you will limit the availability].

Example: "If I arrive home in the evening, then I will give all unhealthy snacks to my neighbour."

In this example, " arrive home in the evening " is the cue and "give all unhealthy snacks to my neighbour " is restricting the availability of the unhealthy snacks.

Other ways to limit the availability could be to:

- Put the snacks out of your home in a very inaccessible place
- Have another person from your household lock the snacks in a cupboard to which you do not have access
- When you have used up the snacks, do not buy any more

Please now write down your if-then plan exactly according to this scheme.
Please note that you can keep to this plan for the next 12 weeks and cannot change it. [open response]

***
Now we come to the third step for the habit change:

Please think about any other preparatory measures needed to implement your plan.

This could be, for example

- set a notification/reminder on your mobile device or write a handwritten note to stop buying new snacks
- Discuss your plans with people close to you (e.g. persons you live with)
- Avoid the supermarket sections with unhealthy snacks
- Don't go shopping when you're hungry

Now please indicate what other actions you would like to take to ensure that you do not encounter unhealthy snacks when your "{cue}" cue occurs.
[open response]

Instructions for intervention phase (**all intervention** groups; day 7)
**Congratulations!**<br><br>You now have an if-then plan to help you stop your unhealthy snack consumption when your cue occurs. You can view this at any time from now in the "My Plan" tab. In this tab you can also see the preparatory steps you set yourself.<br><br>**We now come to the last step for the habit change:**<br><br>From now on, you will be prompted to stop your unhealthy snack consumption using your If-Then Plan when your cue occurs. Please try to consistently implement your plan each time you encounter your chosen cue. It is important for the study that you apply the formulated plan and do not try out any other strategies during the study.<br><br>**Over the next 12 weeks, we ask you to record every encounter with your cue in the app.**<br><br>It is important that you record these encounters as soon as possible after they occur. This only takes a few seconds. You can now record events at any time in the trigger questionnaire on the start page of the app. Please note that you can complete the trigger questionnaire repeatedly (whenever you encounter the trigger). In addition, unlike the evening questionnaire, the trigger questionnaire is available throughout the day.<br><br>**We ask you to test this function immediately after completing this survey.**<br><br>**We also ask you to complete the evening diary questionnaires as before.**<br><br>If you have any questions or comments, please feel free to contact us at habirupt.psy@unibe.ch.

1.4.2 App features

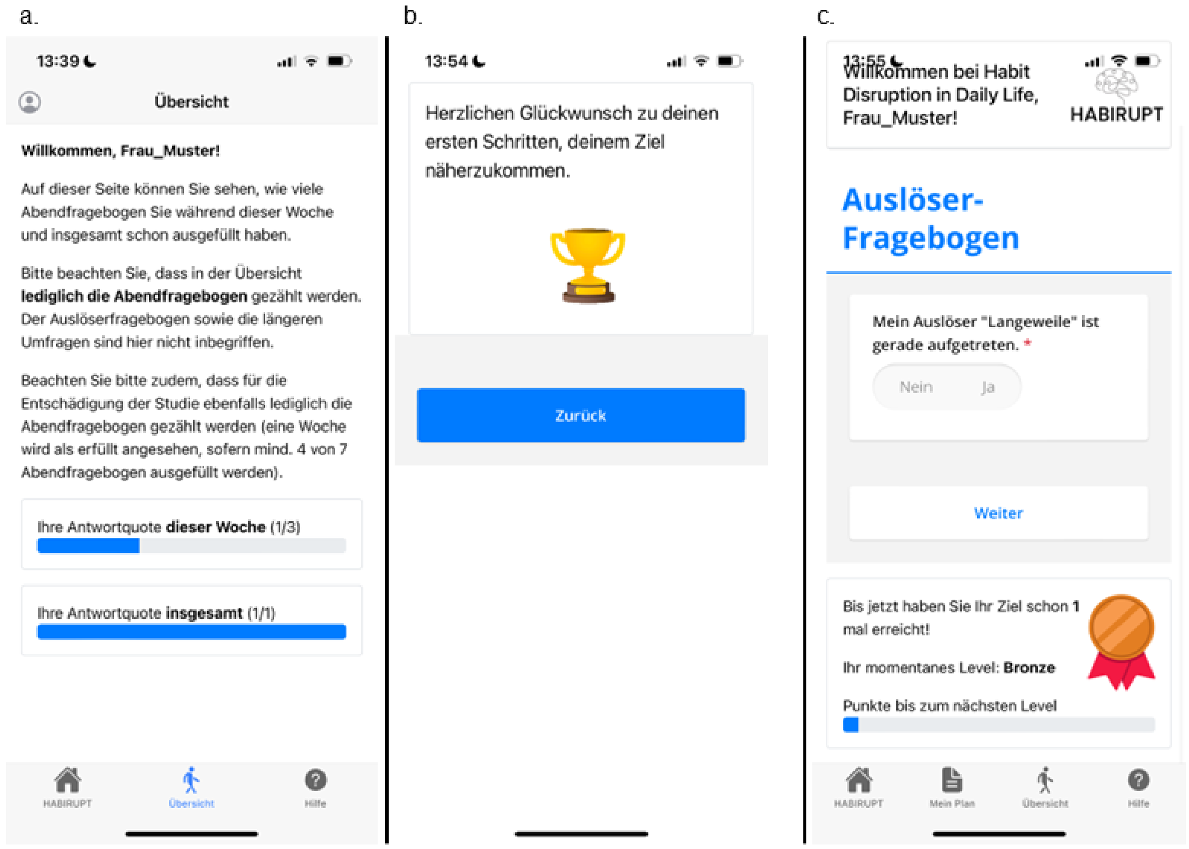

Fig. S1 | Screenshots from smartphone app. Screenshots have been taken by the research team. **a. App overview tab** displays the number of end-of-day questionnaire responses completed for the current week and for the entire study duration with a progress bar. The progress bar was unrelated to reward and could be viewed by all participants. Note that the progress bar values are faulty in the displayed example. **b. Reward message pop-up screen** displaying a reward message and an animated trophy graphic. **c. App homepage tab** during the intervention phase for a participant in the reward condition. The upper half of the tab contains the continuously available event-contingent questionnaire labelled "Auslöser-Fragebogen" (Eng. Cue questionnaire). In the lower half of the tab are displayed the in-app points accumulated for encountering cue without subsequent unhealthy snacking (here 1 point), the accomplishment tier based on accumulated points (here bronze), and a progress bar indicating when the next accomplishment tier (silver, gold or diamond) will be achieved.

Table S2 | Example reward messages used in the study.

Timing (study day)	Reward message
7	Congratulations on your first steps towards your goal.
8	Today you proved your discipline by successfully resisting the temptation of unhealthy snacks. Well done!
18	Brilliant! What you have achieved is down to your own determination alone!
21	Hats off, you did a great job today! You have now already mastered 2 weeks.
38	Impressive! You successfully put your resolution into practice, you can be proud of yourself!
53	Your willpower is simply impressive! Stay committed!
60	60 days have already passed! You have unequivocally showcased your determination!
91	Congratulations to you! You have reached the end of this study duration and have proven that determination and perseverance pays off. These qualities promise great success for your future. Celebrate this achievement, because you have truly earned it!

Note. Reward messages are translated from German; Full list of original reward messages are available online (https://osf.io/z7tby/).

1.5 Methods for intervention fidelity and manipulation check

Intervention fidelity was assessed in relation to strategy and reward. First regarding strategy, in the post-study survey, participants reported of any additional strategies they might have used (subsequently referred to as blended strategy use) to degrade their unhealthy snacking habit beyond the strategy inherent to the experimental manipulation. Also, during data processing, the actual strategies used in the implementation intentions were manually coded as a fidelity check to verify congruence with assigned strategy. These fidelity assessments reflect treatment enactment[6].

Regarding intervention fidelity of reward, the duration of time, in seconds, spent with an in-app pop-up screen displaying a reward message was recorded. This served as an intervention fidelity check for treatment receipt[6]. Regarding the manipulation check of reward, perceived reward was assessed using a single 5-point Likert scale item: "Just now, that I didn't eat any unhealthy snacks in my selected situation felt good" (answer options: not true–completely true) on 12

prespecified occasions (2nd, 5th, 10th, 15th, 21st, 32nd, 45th, 50th, 61st, 78th, 80th, and 88th sequential occasion reward delivery) to participants in a reward condition (see manuscript Fig. 3). The event-contingent perceived reward question was available for participants for 30 minutes after they had recorded avoiding unhealthy snacking in response to a cue encounter.

1.6 Data analysis

1.6.1 Event contingent data processing

The event-contingent data was cleaned as follows (procedure established post-hoc). First entries with missing data in all key variables were removed (k = 2,110 entries removed), along with entries where the cue was reportedly not encountered (contradicts intended event-contingency, k = 1,857 entries removed). Then duplicate entries (defined as entries < 50 seconds apart based on data familiarization) were removed (k = 116 entries removed) by retaining the temporally last entry, while ensuring no data loss (imputing missing data if available in temporally earlier duplicate entry).

1.6.2 Main analysis assumption testing

For ANOVA based analyses, the normality of residuals (Shapiro-Wilk test[7]), and homogeneity of variances (Levene's test[8]) were checked. For ANCOVA based analyses, linearity between covariate and dependent variable (visual inspection of scatter plot), and homogeneity of regression slopes (fitting model with interaction term and testing significance of interaction term) were additionally checked. For analyses predicting rate of change H2.1 (weeks 1 and 2) and H2.2-.3 (weeks 1 and 2), the normality of residuals assumption was violated, for which reason robust ANOVA (H2.1) and robust regression (H2.2-.3) were conducted as sensitivity analyses. For analyses predicting time needed to reach the lower 95% asymptote (H2.4-.6) initial non-normality of residuals was handled with log-transformation of the dependent variable. For analysis predicting the binary outcome of reaching 95% asymptote (H1.4-.6) assumptions of logistic regression were evaluated by checking multicollinearity (variance inflation factor), linearity of logit (Box-Tidwell test[9]), checking for influential observations, and

goodness of fit (Hosmer-Lemeshow test[10]). For H1.5-.6, the Hosmer-Lemeshow test was initially below the threshold for good fit (p < .05). Sensitivity analysis excluding flagged influential cases improved the model fit (p = 0.20), while all predictors remained non-significant. Given the stability of results and the absence of data errors, all cases were retained in the final analysis.

1.7 Statistical software

Within-person asymptotic models were estimated using the 'stats::Ssasymp()' base R function and the 'nls.multstart' R package[11] to iteratively test multiple starting values for each within-person time series. GAMs were estimated using the 'mgcv' R package[12], and the 'gratia' R package[13] was used to obtain the first derivatives. ANOVA and ANCOVA analyses were run with R package 'afex'[14].

1.8 Deviation from protocol

The preregistration stated the perceived reward item would be delivered to all intervention group participants, and not only reward condition participants as conducted. This deviation was made to avoid inducing reward to the non-reward condition participants through enhanced self-monitoring. Further, participants were not excluded from primary analyses based on not affirming plan enactment at least once during the study as stated in the preregistration. This deviation was made because of participants' low uptake of responding to the event-contingent questionnaire, which would have led to a reduction in statistical power if participants were excluded. Sensitivity analysis with reassigned actual strategy group and exclusion of blended strategy use was added post-hoc.

2. Supplementary notes

2.1 Supplementary notes 1: Participant retention and habit strength observations

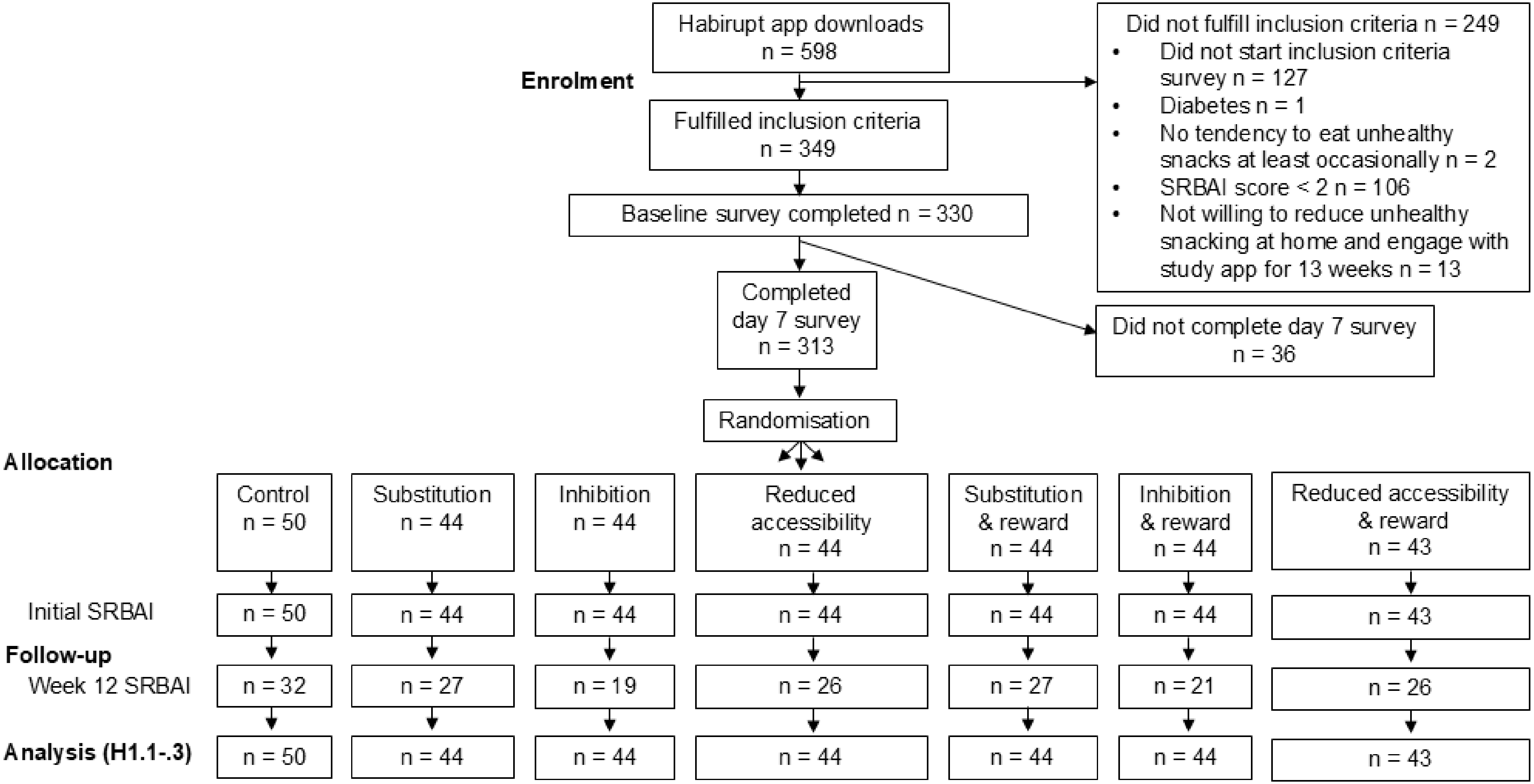

Fig. S2 | Participant flow chart. For group specific sample sizes of other primary analyses see Table S3. SRBAI: Self-Report Behavioural Automaticity Index.

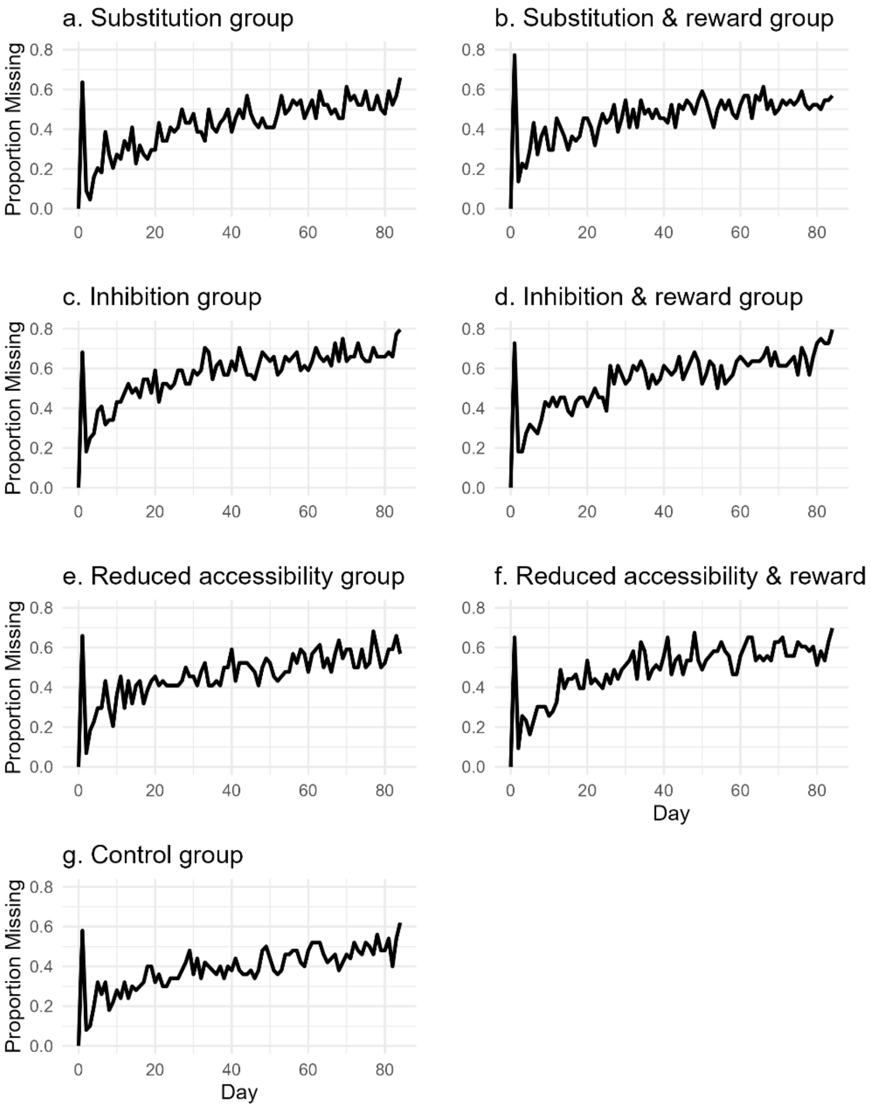

Fig. S3 | Proportion of missing daily habit strength observations by allocated intervention group. Panels a–g present plots for each intervention group as indicated in the individual plot titles. Day ranges from 0-84, where day 0 corresponds to day 7 (initial habit strength measurement) and subsequent days the entire intervention phase. The spike in proportion of missing habit strength observations on day 1 of the intervention phase, was presumably due to a technical issue with the app. Comprehensive heat maps of habit strength values and missing observations are available in the online repository time series data visualizations file (https://osf.io/z7tby/).

2.2 Supplementary notes 2: Within-person habit degradation trajectories and outcomes extracted

Within-person asymptotic models were sequentially deemed invalid (n = 234) first due to missing gaps of observations longer than 21 days (n = 122), then due to displaying an increasing trend (n = 29), and lastly due to poor absolute fit (RMSE > 0.33; n = 83). Regarding generalized additive models (GAMs), in 10 instances person-specific models could not be estimated because only 1 observation was recoded (initial habit strength on day 7), and in 31 instances the time series did not meet the criteria of having at least one observation during the intervention phase week 1, week 2 and time following week 2. In one instance despite containing sufficient data, the person-specific GAM could not be estimated due to a lack of variance (i.e. habit strength remained constant over time); consequently, rate of change equal to zero was imputed for this participant. See the online repository time series data visualization file for plots of all within-person time series and model predictions (https://osf.io/z7tby/).

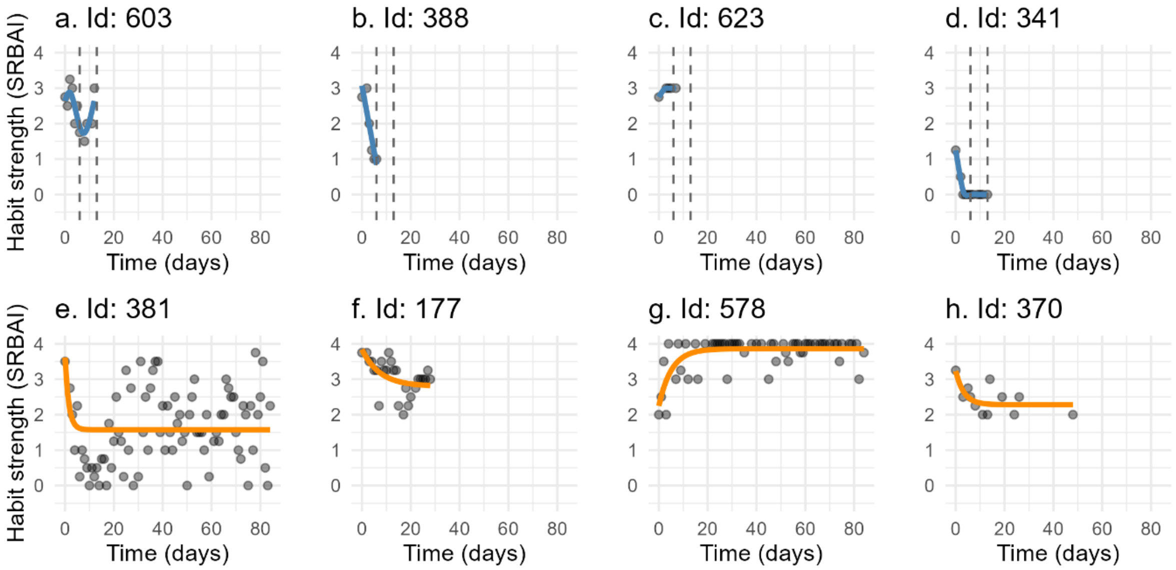

Fig. S4 | GAMs (panels a.-d.) and asymptotic models (panels e.-h.) that did not meet set criteria. Dashed vertical lines (panels a.-d.) indicate the 7th and 14th day of the intervention phase. GAMs were required to contain at least one SRBAI observation during the first week, second week and time following the second week for the rate of change to be calculated for the first and second weeks. Idiographic asymptotic models (panels e.-h.) were deemed invalid estimates if the root-mean-square error was above 0.33 (panel e.), the time series contained a consecutive gap of SRBAI observations longer than 21 days (panels f. and g.), or the trajectory approached an upper asymptote (panel g.); SRBAI: Self-reported behavioural automaticity index; GAMs: generalized additive models.

Table S3 | Descriptive statistics of observed habit strength and indicators of habit degradation by allocated group.

Measure	Intervention group	Reward condition	*n*	Mean	*IQR*
Initial observed habit strength	full sample	not applicable	313	2.63	2.25, 3.00
	control	not applicable	50	2.65	2.25, 3.00
	intervention	not applicable	263	2.58	2.00, 3.25
	inhibition	no reward	44	2.50	2.25, 3.00
	inhibition	reward	44	2.84	2.25, 3.31
	substitution	no reward	44	2.63	2.00, 3.00
	substitution	reward	44	2.78	2.50, 3.00
	reduced accessibility	no reward	44	2.67	2.25, 3.25
	reduced accessibility	reward	43	2.45	2.00, 3.00
Final observed habit strength*	full sample	not applicable	313	1.53	0.94, 2.08
	control	not applicable	50	1.69	0.75, 2.50
	intervention	not applicable	263	1.5	0.95, 2.00
	inhibition	no reward	44	1.63	1.00, 2.09
	inhibition	reward	44	1.65	1.00, 2.25
	substitution	no reward	44	1.41	0.59, 2.00
	substitution	reward	44	1.29	0.63, 1.91
	reduced accessibility	no reward	44	1.42	0.87, 2.00
	reduced accessibility	reward	43	1.60	1.00, 2.11
Week 12 average habit strength	full sample	not applicable	178	1.34	0.59, 2.00
	control	not applicable	32	1.65	0.75, 2.27
	intervention	not applicable	146	1.27	0.52, 2.00
	inhibition	no reward	19	1.50	0.75, 2.02
	inhibition	reward	21	1.18	0.35, 2.00
	substitution	no reward	27	1.41	0.56, 2.06
	substitution	reward	27	0.97	0.21, 1.45
	reduced accessibility	no reward	26	1.29	0.64, 1.85
	reduced accessibility	reward	26	1.35	0.93, 1.96

Measure	Intervention group	Reward condition	*n*	Mean	*IQR*
Frequency of reaching and time needed to reach 95% of lower asymptote**	full sample	not applicable	66	21.79	6.25, 27.00
	control	not applicable	12	13.58	1.75, 23.50
	intervention	not applicable	54	23.61	8.25, 31.50
	inhibition	no reward	8	29.50	9.25, 44.25
	inhibition	reward	7	24.71	12.50, 25.50
	substitution	no reward	10	13.90	5.25, 24.00
	substitution	reward	9	26.89	11.00, 26.00
	reduced accessibility	no reward	10	33.00	13.00, 48.25
	reduced accessibility	reward	10	15.50	5.25, 22.25
Week 1 daily average habit strength rate of change	full sample	not applicable	250	-0.07	-0.11, -0.01
	control	not applicable	42	-0.03	-0.07, 0.00
	intervention	not applicable	208	-0.07	-0.12, -0.01
	inhibition	no reward	30	-0.06	-0.10. 0.00
	inhibition	reward	36	-0.07	-0.11, -0.01
	substitution	no reward	38	-0.09	-0.13, -0.01
	substitution	reward	34	-0.09	-0.12, -0.02
	reduced accessibility	no reward	37	-0.08	-0.13, -0.02
	reduced accessibility	reward	33	-0.05	-0.08, -0.01
Week 2 daily average habit strength rate of change	full sample	not applicable	250	-0.03	-0.06, 0.00
	control	not applicable	42	-0.02	-0.02, 0.00
	intervention	not applicable	208	-0.03	-0.06, 0.00
	inhibition	no reward	30	-0.03	-0.07, 0.00
	inhibition	reward	36	-0.03	-0.06, 0.00
	substitution	no reward	38	-0.03	-0.07, 0.00
	substitution	reward	34	-0.04	-0.06, -0.02
	reduced accessibility	no reward	37	-0.05	-0.06, -0.01
	reduced accessibility	reward	33	-0.03	-0.05, 0.00

Note. *IQR*: Interquartile range (25%, 75%). *Based on the average week 12 habit strength score, or the last available habit strength observation carried forward if no observations were available for week 12; **Frequency depicted with n and time in days depicted with mean and *IQR*.

2.3 Supplementary notes 3: Main analysis

Concerning the covariate of primary analyses, initial habit strength was a significant covariate of final habit strength in the H1.1 model ($F(1, 310) = 45.10$, $p < .001$, generalized eta squared (*ges*) = .127) and in the H1.2-.3 model ($F(1, 256) = 30.79$, $p < .001$, *ges* = .107). In contrast, initial habit strength was not a significant covariate in model H1.4 ($z(1) = -1.703$, $p = 0.089$, odds ratio = 0.732), model H1.5-.6 ($\chi^2(1) = 2.479$, $p = 0.115$, odds ratio = 0.717), model H2.4 ($F(1, 63) = 1.87$, $p = 0.177$, *ges* = 0.029), or in model H2.5-.6 ($F(1, 47) = 0.07$, $p = 0.787$, *ges* = 0.002).

Table S4 | Pairwise comparisons from logistic regression results for the likelihood of reaching 95% of the lower asymptote.

Model	Contrast	*OR (SE)*	95% *CI*	*p*
H1.4	control / intervention	1.19 (0.44)	[0.58, 2.45]	0.631
H1.5-1.6	No reward: reduced accessibility / inhibition*	1.41 (0.75)	[0.40, 4.94]	0.798
	No reward: reduced accessibility / substitution*	1.02 (0.52)	[0.31, 3.38]	0.999
	No reward: inhibition / substitution*	0.72 (0.39)	[0.21, 2.53]	0.817
	Reward: reduced accessibility / inhibition*	1.42 (0.79)	[0.39, 5.22]	0.803
	Reward: reduced accessibility / substitution*	1.05 (0.56)	[0.31, 3.62]	0.995
	Reward: inhibition / substitution*	0.74 (0.42)	[0.20, 2.75]	0.855
	Reduced accessibility: no reward / reward	1.04 (0.54)	[0.38, 2.86]	0.932
	Inhibition: no reward / reward	1.05 (0.61)	[0.34, 3.25]	0.928
	Substitution: no reward / reward	1.08 (0.57)	[0.39, 3.01]	0.882

Note. *OR*: Odds ratio. Odds ratios are estimated marginal means back-transformed from the log odds scale; *SE*: Standard error; *CI*: confidence interval. *Confidence intervals and *p*-values adjusted with Tukey methods for comparing a family of 3 estimates.

2.4 Supplementary notes 4: Intervention fidelity and manipulation check

Manual coding of strategies used in implementation intentions revealed inconsistent adherence to intervention arm specific guidelines. Specifically, 51% (45/88) of participants assigned to an inhibition strategy group formulated implementation intentions aiming to inhibit habitual unhealthy snacking, and 45% (39/87) of participants assigned to a reduced accessibility strategy group formulated implementation intentions aiming to reduce accessibility of unhealthy snacks. Adherence was higher in the substitution group, where 98% (86/88) participants formulated implementation intentions aiming at replacing habitual unhealthy snacking with an alternative response. When further accounting for participants' self-reported additional strategy use based on post-study survey responses, adherence decreased further. Specifically, when excluding participants who reported usage of unassigned strategies adherence rates were 50% (25 / 50) for the control group, 31% (27 / 88) for inhibition groups, 48% (42 / 88) for substitution groups, and 25% (22 / 87) for reduced accessibility groups (see Supplementary Table S5).

Intervention fidelity of reward is first assessed in light of responding frequency to the event-contingent questionnaire, as this served as the trigger for reward delivery. For the entire sample over the course of the study (excluding control group participants) 2,311 cue encounters were recorded across 216 participants (i.e. 47 (18%) intervention group participants never recorded a cue encounter). Event-contingent entries of cue-encounters decreased with time, as 50% (1157 / 2311) of cue encounters were recorded by day 22 of the intervention phase. In 57% (1,324 / 2,311) of cue encounters, no subsequent snacking occurred. In total 158 participants recorded no snacking following a cue encounter at least once, of which 91 participants were in a reward condition (entailing 920 observations). These 920 observations were triggers for reward delivery, but it cannot be confirmed that reward was delivered on all intended occasions. The duration of viewing a reward message was available for 639 observations across 85 participants, for which the mean value was 2.7 seconds (interquartile range: 1.3, 3.3 seconds).

Regarding the manipulation check of reward, perceived reward was evaluated in total 89 times across 49 participants, for which the mean value was 3.3 (median = 4; interquartile range: 3, 4) suggesting high perceived reward. 50% (45 / 89) of perceived reward observations were recorded by day 12 of the intervention phase, and 75% (67 / 89) were recorded by day 30.

2.5 Supplementary notes 5: Sensitivity analyses

Sensitivity analyses addressing magnitude of change in habit strength (H1.1-.3) broadly supported the primary findings (see Supplementary Table S5 for descriptive statistics and Table S6 for sensitivity analysis results). However, in one of the four sensitivity analysis comparing intervention and control groups, there was a significant intervention effect when excluding participants without habit strength observations in the final week of the study. Specifically, the magnitude of change was significantly greater in the intervention group compared to control ($F(1, 175) = 4.54$, $p = 0.034$, *ges* = 0.025). However, as this result was not evident in any other analyses conducted, this is not considered worthy of further interpretation. Additionally, when reassignment to actual strategy use was modelled (H1.2-.3), a significant interaction emerged ($F(2, 133) = 3.35$, $p = 0.038$, *ges* = 0.048), suggesting a smaller magnitude of change for the reduced accessibility strategy when combined with reward compared to without reward. This interaction remained significant when additionally excluding participants without observations during the last week of the study ($F(2, 246) = 3.25$, $p = 0.040$, *ges* = 0.026). Descriptively, this interaction was somewhat visible in the primary analysis as well (see manuscript Fig. 6 panel b). However, as these interaction results are based on a small number of participants, it is not considered worthy of further interpretation.

Table S5 | Descriptive statistics of variables used in sensitivity analyses.

	Overall (N = 313)	Control (n = 50)	Inhibition (n = 44)	Inhibition & reward (n = 44)	Substitution (n = 44)	Substitution & reward (n = 44)	Reduced accessibility (n = 44)	Reduced accessibility & reward (n = 43)
Desirable responding: self-deception enhancement	3.72 (3.33, 4.33)	3.69 (3.33, 4.33)	3.73 (3.33, 4.33)	3.95 (3.33, 4.67)	3.72 (3.33, 4.33)	3.64 (3.17, 4.00)	3.70 (3.33, 4.00)	3.63 (3.33, 4.17)
(missing)	9	0	0	2	1	0	3	3
Desirable responding: impression management	4.27 (3.67, 5.00)	4.19 (3.67, 4.67)	4.36 (3.67, 5.00)	4.25 (3.67, 5.00)	4.36 (3.67, 5.00)	4.55 (4.00, 5.33)	4.11 (3.33, 5.00)	4.06 (3.33, 5.00)
(missing)	8	0	0	2	0	0	3	3
Intention*	3.27 (3.00, 4.00)	3.12 (2.50, 4.00)	3.34 (3.00, 4.00)	3.31 (3.00, 4.00)	3.31 (3.00, 4.00)	3.30 (3.00, 4.00)	3.28 (3.00, 3.75)	3.24 (3.00, 4.00)
Reassigned actual strategy								
Control	50 (17%)	50 (100%)	0 (0%)	0 (0%)	0 (0%)	0 (0%)	0 (0%)	0 (0%)
Inhibition	55 (18%)	0 (0%)	21 (48%)	24 (57%)	1 (2.3%)	0 (0%)	5 (13%)	4 (10%)
Substitution	157 (52%)	0 (0%)	21 (48%)	18 (43%)	43 (98%)	43 (100%)	14 (35%)	18 (45%)
Reduced accessibility	41 (14%)	0 (0%)	2 (4.5%)	0 (0%)	0 (0%)	0 (0%)	21 (53%)	18 (45%)
None of above	10	0	0	2	0	1	4	3
Reassigned actual strategy, excluding blended strategy use								
Control	25 (16%)	25 (100%)	0 (0%)	0 (0%)	0 (0%)	0 (0%)	0 (0%)	0 (0%)
Inhibition	33 (21%)	0 (0%)	15 (54%)	12 (55%)	1 (4.8%)	0 (0%)	1 (5.3%)	4 (19%)
Substitution	78 (49%)	0 (0%)	13 (46%)	10 (45%)	20 (95%)	22 (100%)	6 (32%)	7 (33%)
Reduced accessibility	22 (14%)	0 (0%)	0 (0%)	0 (0%)	0 (0%)	0 (0%)	12 (63%)	10 (48%)
None of above / blended	155	25	16	22	23	22	25	22

Note. Desirable responding and intention reported with mean (interquartile range: 25%, 75%); Desirable responding measured with the Balanced Inventory of Desirable Responding (BIDR[15]; score range 0-6); *Intention to prevent unhealthy snacking at cue encounter for next 12 weeks. Intention measured on day 7 (score range 0-6); Reassigned strategy reported with n (%).

Table S6 | Estimated marginal means and test statistics of sensitivity analyses with magnitude of change as outcome (H1.1-.3).

Sensitivity analysis	Intervention group	Reward condition	*n*	Adjusted mean (*SE*)	95% *CI*	Test statistic
H1.1-.3 models: 1. Excluding participants with no SRBAI observations during last week	control	not applicable	32	1.67 (0.17)	[1.33, 2.00]	*F*(1, 175) = 4.54, *p* = **0.034**, *ges* = 0.025
	intervention	not applicable	146	1.27 (0.08)	[1.11, 1.43]	
	inhibition	no reward	19	1.54 (0.21)	[1.13, 1.96]	Intervention group: *F*(2, 139) = 0.79, *p* = 0.454, *ges* = 0.011 Reward condition: *F*(1, 139) = 3.04, *p* = 0.084, *ges* = 0.021 Interaction: *F*(2, 139) = 1.40, *p* = 0.250, *ges* = 0.020
	inhibition	reward	21	1.12 (0.20)	[0.73, 1.52]	
	substitution	no reward	27	1.39 (0.18)	[1.04, 1.73]	
	substitution	reward	27	0.92 (0.18)	[0.57, 1.27]	
	reduced accessibility	no reward	26	1.32 (0.18)	[0.97, 1.68]	
	reduced accessibility	reward	26	1.41 (0.18)	[1.05, 1.76]	
H1.1-.3 models: 2. Covariates added: intention strength, BMI, and desirable responding	control	not applicable	50	1.67 (0.13)	[1.41, 1.92]	*F*(1, 297) = 1.41, *p* = 0.237, *ges* = 0.005
	intervention	not applicable	254	1.50 (0.06)	[1.38, 1.61]	
	inhibition	no reward	44	1.73 (0.14)	[1.46, 2.00]	Intervention group: *F*(2, 243) = 2.35, *p* = 0.098, *ges* = 0.020 Reward condition: *F*(1, 243) = 0.12, *p* = 0.733, *ges* < 0.001 Interaction: *F*(2, 243) = 1.81, *p* = 0.166, *ges* = 0.015
	inhibition	reward	42	1.51 (0.14)	[1.23, 1.79]	
	substitution	no reward	43	1.41 (0.14)	[1.14, 1.68]	
	substitution	reward	44	1.24 (0.14)	[0.97, 1.51]	
	reduced accessibility	no reward	41	1.40 (0.14)	[1.12, 1.68]	
	reduced accessibility	reward	40	1.67 (0.15)	[1.38, 1.95]	
H1.1-.3 models: 1. & 2. combined	control	not applicable	32	1.51 (0.17)	[1.18, 1.85]	*F*(1, 166) = 1.28, *p* = 0.260, *ges* = 0.008
	intervention	not applicable	141	1.30 (0.08)	[1.14, 1.46]	
	inhibition	no reward	19	1.63 (0.21)	[1.22, 2.05]	Intervention group: *F*(2, 130) = 0.38, *p* = 0.684, *ges* = 0.006 Reward condition: *F*(1, 130) = 3.75, *p* = 0.055, *ges* = 0.020 Interaction: *F*(2, 130) = 1.81, *p* = 0.168, *ges* = 0.027
	inhibition	reward	20	1.02 (0.21)	[0.62, 1.43]	
	substitution	no reward	27	1.38 (0.17)	[1.04, 1.72]	
	substitution	reward	27	0.99 (0.18)	[0.64, 1.34]	
	reduced accessibility	no reward	24	1.27 (0.19)	[0.91, 1.64]	
	reduced accessibility	reward	24	1.38 (0.18)	[1.01, 1.74]	

Table S6 (continued) | Estimated marginal means and test statistics of sensitivity analyses with magnitude of change as outcome (H1.1-.3).

Sensitivity analysis	Intervention group	Reward condition	*n*	Adjusted mean (*SE*)	95% *CI*	Test statistic
H1.2-.3 model: Reassigned actual strategy group*	inhibition	no reward	27	1.62 (0.18)	[1.27, 1.97]	Strategy group: *F*(2, 246) = 0.00, *p* = 0.982, *ges* < 0.001 Reward condition: *F*(1, 246) = 0.39, *p* = 0.535, *ges* = 0.002 Interaction: *F*(2, 246) = 3.25, *p* = **0.040**, *ges* = 0.026
	inhibition	reward	28	1.34 (0.18)	[1.00, 1.69]	
	substitution	no reward	78	1.56 (0.11)	[1.35, 1.77]	
	substitution	reward	79	1.46 (0.11)	[1.25, 1.66]	
	reduced accessibility	no reward	23	1.18 (0.19)	[0.80, 1.56]	
	reduced accessibility	reward	18	1.82 (0.22)	[1.39, 2.26]	
H1.2-.3 model: 1. & reassigned actual strategy group*	inhibition	no reward	10	1.47 (0.29)	[0.89, 2.05]	Strategy group: *F*(2, 133) = 0.19, *p* = 0.831, *ges* = 0.003 Reward condition: *F*(1, 133) = 0.27, *p* = 0.605, *ges* = 0.002 Interaction: *F*(2, 133) = 3.35, *p* = **0.038**, *ges* = 0.048
	inhibition	reward	14	0.91 (0.25)	[0.42, 1.39]	
	substitution	no reward	46	1.50 (0.14)	[1.23, 1.76]	
	substitution	reward	46	1.13 (0.14)	[0.86, 1.39]	
	reduced accessibility	no reward	12	0.93 (0.26)	[0.41, 1.45]	
	reduced accessibility	reward	12	1.57 (0.26)	[1.05, 2.09]	
H1.1-.3 models: 1. & reassigned actual strategy group & excluding blended strategy use	control	not applicable	10	1.86 (0.28)	[1.29, 2.43]	*F*(1, 44) = 3.89, *p* = 0.055, *ges* = 0.081
	intervention	not applicable	37	1.23 (0.15)	[0.94, 1.53]	
	inhibition	no reward	4	1.10 (0.47)	[0.15, 2.06]	Strategy group: *F*(2, 30) = 0.19, *p* = 0.829, *ges* = 0.012 Reward condition: *F*(1, 30) = 0.30, *p* = 0.590, *ges* = 0.010 Interaction: *F*(1, 30) = 0.36, *p* = 0.566, *ges* = 0.012
	inhibition	reward	4	1.08 (0.47)	[0.13, 2.03]	
	substitution	no reward	10	1.41 (0.29)	[0.81, 2.01]	
	substitution	reward	12	1.22 (0.27)	[0.68, 1.77]	
	reduced accessibility	no reward	3	0.80 (0.55)	[-0.32, 1.91]	
	reduced accessibility	reward	4	1.58 (0.46)	[0.63, 2.53]	

Note. *Only reassigning actual strategy use (and not also excluding blended strategy use) cannot be done for control group, because reassignment is based on implementation intentions which control group participants did not formulate; *SE*: Standard error; *CI*: Confidence interval; *ges*: generalized eta squared; SRBAI: Self-reported behavioural automaticity index; BMI: body mass index.

Sensitivity analysis addressing likelihood to reach asymptote (H1.4.-6) were congruent with primary analyses as they did not provide evidence to suggest a group difference (Supplementary Table S7). To elucidate, in these analyses participant's strategy used was reassigned based on the actual strategy used in the implementation intention and participants who reported blended use of several strategies were removed.

Sensitivity analyses addressing week 1 rate of change supported primary analyses. Firstly, sensitivity analysis using robust ANOVA supported the findings of a significant effect of intervention group status being associated to a faster rate of change compared to the control group for week 1 ($F(1, 49.19) = 8.12$, $p = 0.006$, bootstrap confidence interval (*CI*) = [0.03, 0.58], explanatory measure of effect size = 0.32). Additionally, findings suggesting a faster rate of change in the intervention compared to control group remained significant (Supplementary Table S8) when participants were reassigned based on actual strategy use and those with blended strategy use were excluded ($F(1, 98) = 5.65$, $p = 0.019$, *ges* = 0.054). Lastly, in congruence with primary analyses, results concerning comparison of intervention groups (or actual strategy use) and reward condition remained statistically insignificant in sensitivity analysis for week 1 rate of change (Supplementary Table S8).

Sensitivity analyses addressing week 2 rate of change findings were less consistent. When comparing intervention and control groups (H2.1), sensitivity analysis using robust ANOVA supported the findings of a significant effect of intervention group status being associated to a faster rate of change compared to the control group for week 2 ($F(1, 40.12) = 7.67$, $p = 0.008$, bootstrap *CI* = [0.03, 0.57], explanatory measure of effect size = 0.33). However, the main effect of intervention group was not replicated (Supplementary Table S8) when modelling reassigned actual strategy and excluding blended strategy use ($F(1, 98) = 1.30$, $p = 0.257$, *ges* = 0.013).

When comparing intervention groups and reward condition (H2.2-.3), sensitivity analysis using robust regression supported the findings of a non-significant effect for week 1 and week 2 based

on t-values. In the week 1 analysis, the coefficient for reward was the largest in magnitude among predictors (t(202) = 1.38, estimate = 0.025, SE = 0.02). In the week 2 analysis, the coefficient for the strategy group inhibition was the largest in magnitude among predictors (t(202) = 1.29, estimate = 0.013, SE = 0.01). In turn sensitivity analyses with reassigned actual strategy and excluding blended strategy use, a significant main effect for strategy (F(2, 77) = 3.31, p = 0.042, ges = 0.079), reward (F(1, 77) = 6.50, p = 0.013, ges = 0.078) and interaction (F(2, 77) = 7.02, p = 0.002, ges = 0.154) emerged (Supplementary Table S7). However, as these results are based on a small number of participants and do not replicate in any other analyses conducted, they are not considered worthy of further interpretation.

Table S7 | Estimated marginal means and test statistics of sensitivity analyses with likelihood of reaching asymptote as outcome (H1.4-.6).

Sensitivity analyses	Intervention group	Reward condition	*n*	Adjusted mean (*SE*)	95% *CI*	Test statistic
Reassigned actual strategy group & excluding blended strategy use	control	not applicable	25	0.22 (0.08)	[0.10, 0.43]	z(1) = 1.682, p = 0.093
	active	not applicable	133	0.10 (0.03)	[0.06, 0.17]	
	inhibition	no	17	0.12 (0.08)	[0.03, 0.37]	Intervention group: $\chi^2(2) = 0.086$, p = 0.958<br>Reward condition: $\chi^2(1) = 1.683$, p = 0.195<br>Interaction: $\chi^2(2) = 4.433$, p = 0.109
	inhibition	yes	16	0.00 (0.00)	[0.00, 1.00]	
	substitution	no	39	0.1 (0.05)	[0.04, 0.24]	
	substitution	yes	39	0.11 (0.05)	[0.04, 0.25]	
	reduced accessibility	no	12	0.08 (0.08)	[0.01, 0.42]	
	reduced accessibility	yes	10	0.30 (0.14)	[0.10, 0.62]	

Note. Only reassigning actual strategy use (and not also excluding blended strategy use) cannot be done for control group, because reassignment is based on implementation intentions which control group participants did not formulate; *SE*: Standard error; *CI*: Confidence interval.

Table S8 | Estimated marginal means and test statistics of sensitivity analyses with rate of change as outcome (H2.1-.3).

Sensitivity analysis	Intervention group	Reward condition	*n*	Adjusted mean (*SE*)	95% *CI*	Test statistic
H2.1-.3 models: Reassigned actual strategy group & excluding blended strategy use Week 1	control	not applicable	17	-0.02 (0.02)	[-0.07, 0.03]	$F(1, 98) = 5.65$, $p =$ **0.019**, *ges* = 0.054
	intervention	not applicable	83	-0.08 (0.01)	[-0.10, -0.06]	
	inhibition	no reward	9	-0.09 (0.03)	[-0.16, -0.02]	Strategy group: $F(2, 77) = 0.05$, $p = 0.951$, *ges* = 0.001<br>Reward condition: $F(1, 77) = 2.81$, $p = 0.097$, *ges* = 0.035<br>Interaction: $F(2, 77) = 2.06$, $p = 0.134$, *ges* = 0.058
	inhibition	reward	10	-0.06 (0.03)	[-0.13, 0.00]	
	substitution	no reward	26	-0.07 (0.02)	[-0.11, -0.03]	
	substitution	reward	25	-0.09 (0.02)	[-0.13, -0.04]	
	reduced accessibility	no reward	7	-0.15 (0.04)	[-0.23, -0.07]	
	reduced accessibility	reward	6	-0.03 (0.04)	[-0.11, 0.06]	
H2.1-.3 models: Reassigned actual strategy group & excluding blended strategy use Week 2	control	not applicable	17	-0.02 (0.01)	[-0.04, 0.00]	$F(1, 98) = 1.30$, $p = 0.257$, *ges* = 0.013
	intervention	not applicable	83	-0.03 (0.01)	[-0.04, -0.02]	
	inhibition	no reward	9	-0.03 (0.01)	[-0.06, -0.01]	Strategy group: $F(2, 77) = 3.31$, $p =$ **0.042**, *ges* = 0.079<br>Reward condition: $F(1, 77) = 6.50$, $p =$ **0.013**, *ges* = 0.078<br>Interaction: $F(2, 77) = 7.02$, $p =$ **0.002**, *ges* = 0.154
	inhibition	reward	10	-0.02 (0.01)	[-0.04, 0.00]	
	substitution	no reward	26	-0.02 (0.01)	[-0.04, -0.01]	
	substitution	reward	25	-0.04 (0.01)	[-0.05, -0.02]	
	reduced accessibility	no reward	7	-0.10 (0.01)	[-0.13, -0.07]	
	reduced accessibility	reward	6	-0.02 (0.02)	[-0.05, 0.01]	
H2.2-.3 week 1 model: intention strength covariate added*	inhibition	no reward	30	-0.06 (0.02)	[-0.09, -0.02]	Intervention group: $F(2, 201) = 1.09$, $p = 0.337$, *ges* = 0.011<br>Reward condition: $F(1, 201) = 0.05$, $p = 0.835$, *ges* < 0.001<br>Interaction: $F(2, 201) = 0.97$, $p = 0.382$, *ges* = 0.010
	inhibition	reward	36	-0.08 (0.02)	[-0.11, -0.05]	
	substitution	no reward	38	-0.09 (0.02)	[-0.12, -0.06]	
	substitution	reward	34	-0.09 (0.02)	[-0.12, -0.05]	
	reduced accessibility	no reward	37	-0.08 (0.02)	[-0.11, -0.05]	
	reduced accessibility	reward	33	-0.05 (0.02)	[-0.09, -0.02]	
H2.2-.3 week 2 model: intention strength covariate added*	inhibition	no reward	30	-0.02 (0.01)	[-0.04, -0.01]	Intervention group: $F(2, 201) = 0.56$, $p = 0.571$, *ges* = 0.006<br>Reward condition: $F(1, 201) = 0.02$, $p = 0.891$, *ges* < 0.001<br>Interaction: $F(2, 201) = 2.42$, $p = 0.091$, *ges* = 0.024
	inhibition	reward	36	-0.03 (0.01)	[-0.05, -0.02]	
	substitution	no reward	38	-0.03 (0.01)	[-0.04, -0.02]	
	substitution	reward	34	-0.04 (0.01)	[-0.05, -0.02]	
	reduced accessibility	no reward	37	-0.05 (0.01)	[-0.06, -0.03]	
	reduced accessibility	reward	33	-0.03 (0.01)	[-0.04, -0.01]	

Note. *Sensitivity analysis conducted to account for missingness pattern identified; *SE*: Standard error; *CI*: Confidence interval; *ges*: generalized eta squared.

Supplementary references

1. Edgren, R., Baretta, D. & Inauen, J. The temporal trajectories of habit decay in daily life: An intensive longitudinal study on four health-risk behaviors. *Appl. Psychol. Health Well-Being* aphw.12612 (2024). https://doi.org/10.1111/aphw.12612
2. Lakens, D. & Caldwell, A. R. Simulation-Based Power Analysis for Factorial Analysis of Variance Designs. *Adv. Methods Pract. Psychol. Sci.* **4**, 251524592095150 (2021). https://doi.org/10.1177/2515245920951503
3. OpenAI. ChatGPT. Version June 2025 version [Large language model] (2025). https://chatgpt.com/
4. Ryan, R. M. Control and information in the intrapersonal sphere: An extension of cognitive evaluation theory. *J. Pers. Soc. Psychol.* **43**, 450–461 (1982). https://doi.org/10.1037/0022-3514.43.3.450
5. Ryan, R. M., Mims, V. & Koestner, R. Relation of reward contingency and interpersonal context to intrinsic motivation: A review and test using cognitive evaluation theory. *J. Pers. Soc. Psychol.* **45**, 736–750 (1983). https://doi.org/10.1037/0022-3514.45.4.736
6. Hankonen, N. Participants' enactment of behavior change techniques: a call for increased focus on what people do to manage their motivation and behavior. *Health Psychol. Rev.* **15**, 185–194 (2021). https://doi.org/10.1080/17437199.2020.1814836
7. Royston, J. P. An Extension of Shapiro and Wilk's W Test for Normality to Large Samples. *Appl. Stat.* **31**, 115 (1982). https://doi.org/10.2307/2347973
8. Levene, H. Robust tests for equality of variances. in *Contributions to Probability and Statistics (I. Olkin, ed.)* 278–292 (Stanford Univ. Press, Palo Alto, CA, 1960).
9. Box, G. E. P. & Tidwell, P. W. Transformation of the Independent Variables. *Technometrics* **4**, 531–550 (1962). https://doi.org/10.1080/00401706.1962.10490038

10. Hosmer, D. W. & Lemeshow, S. *Applied Logistic Regression*. (Wiley, 2000). https://doi.org/10.1002/0471722146

11. Padfield, D. & Granville, M. nls.multstart: Robust Non-Linear Regression using AIC Scores (Version R package version 1.2.0.). (2020). https://CRAN.R-project.org/package=nls.multstart

12. Wood, S. N. Thin Plate Regression Splines. *J. R. Stat. Soc. Ser. B Stat. Methodol.* **65**, 95–114 (2003). https://doi.org/10.1111/1467-9868.00374

13. Simpson, G. L. gratia: Graceful ggplot-Based Graphics and Other Functions for GAMs Fitted using mgcv (Version R package version 0.10.0). (2024). https://gavinsimpson.github.io/gratia/

14. Singmann, H., Bolker, B., Westfall, J., Aust, F. & Ben-Shachar, M. afex: Analysis of Factorial Experiments (Version R package version 1.4-1.). (2024). https://CRAN.R-project.org/package=afex

15. Winkler, N., Kroh, M. & Spiess, M. *Entwicklung einer deutschen Kurzskala zur zweidimensionalen Messung von sozialer Erwünschtheit* [Development of a German short scale for two-dimensional measurement of social desirability]. Discussion Papers No. 579 (Deutsches Institut für Wirtschaftsforschung (DIW), Berlin, 2006). http://hdl.handle.net/10419/18472
